# Individualizing the Oncological Treatment of Patients With Metastatic Non–Clear Cell Renal Cell Carcinoma by Using Gene Sequencing and Patient-Reported Outcomes: Protocol for the INDIGO Study

**DOI:** 10.2196/36632

**Published:** 2022-09-15

**Authors:** Ida Marie Lind Rasmussen, Anne Vest Soerensen, Anne Kirstine Møller, Gitte Fredberg Persson, Jesper Andreas Palshof, Gry Assam Taarnhøj, Helle Pappot

**Affiliations:** 1 Department of Oncology Copenhagen University Hospital Herlev Herlev Denmark; 2 Department of Clinical Medicine University of Copenhagen København Denmark; 3 Department of Oncology Rigshospitalet København Denmark

**Keywords:** patient-reported outcome, electronic patient-reported outcome, renal cell carcinoma, non–clear cell renal cell carcinoma, health-related quality of life, oncology, targeted therapy, precision medicine, eHealth, outcome, patient-reported

## Abstract

**Background:**

No phase 3 studies have yet been conducted for patients with non–clear cell (CC) renal cell carcinoma (RCC) exclusively due to the rare occurrence of the disease and the heterogenicity in tumor morphology. Consequently, there is no evidence of the optimal treatment, and new approaches are needed. One approach is individualizing treatment based on the gene sequencing of tumor tissue. Additionally, recent studies involving the patient-reported outcomes (PROs) of patients treated for metastatic cancer have shown significant benefits for quality of life, median overall survival, and overall survival. The use of gene sequencing and PROs can be of great importance to patients with rare cancer types, including patients with non-CC RCC, and should be investigated in clinical trials, especially for cases where evidence based on phase 3 studies is difficult to obtain.

**Objective:**

We describe the INDIGO study, in which patients, based on gene analyses, will be allocated into 4 treatment arms containing 14 treatments and use electronic PROs. We aim to improve the treatment of patients with non-CC RCC. The end points in the study will be the overall response rate (complete and partial) in the total patient population, which will be based on the RECIST (Response Evaluation Criteria in Solid Tumors) version 1.1 criteria, and the time to treatment failure.

**Methods:**

INDIGO is a prospective phase 2 trial, and 30 patients will be enrolled. The patients will receive systemic treatment based on genetic analyses of their tumor tissue. All patients will receive electronic questionnaires in a dedicated app—a questionnaire regarding symptoms and side effects and another regarding health-related quality of life. Depending on the treatment regimen, the patients will be seen by a medical doctor every third, fourth, or sixth week, and the effect of the systemic treatment will be evaluated every 6 weeks via a computed tomography scan. The study has been approved by the Danish Medicines Agency and the National Committee on Health Research Ethics (approval number: H-19041833), complies with good clinical practice guidelines, follows the General Data Protection Regulation, and is registered at the Capital Region of Denmark.

**Results:**

Recruitment started in March 2020, and at the time of submitting this paper (June 2022), a total of 9 patients have been enrolled.

**Conclusions:**

We aim to explore methods for improving the treatment outcomes of patients with non-CC RCC, and the INDIGO study will contribute further data on personalized medicine for rare types of RCC and provide new knowledge on the active use of electronic PROs.

**Trial Registration:**

ClinicalTrials.gov NCT04644432, https://clinicaltrials.gov/ct2/show/NCT04644432 ; European Union Drug Regulating Authorities Clinical Trials Database 2019-001316-38, https://tinyurl.com/2p8mb4aw

**International Registered Report Identifier (IRRID):**

DERR1-10.2196/36632

## Introduction

### Renal Cell Carcinoma

Renal cell carcinoma (RCC) accounts for about 80% of all renal tumors, and the age of onset is typically 60 to 70 years. The majority of patients have clear cell (CC) histology, but 20% have another histology, and this group is referred to as *patients with non-CC RCC* [1]. CC histology is mostly characterized by a von Hippel-Lindau gene defect, whereas non-CC histology comprises different subtypes that each have individual morphological and genetic characteristics [2].

There is no evidence of the optimum treatment of non-CC RCC. No phase 3 studies have been conducted for patients with non-CC RCC exclusively (ie, not for the individual subtype or for the whole group). Data are available from subgroup analyses and the expanded access programs of large studies [1,3-5]. Generally, patients with non-CC RCC have poorer prognoses than those of patients with CC RCC, with an overall survival of 12.8 versus 22.3 months and a time to treatment failure (TTF) of 4.2 versus 7.8 months [2]. The overall response rate (ORR) has been reported in the range of 10% to 27% for patients with non-CC RCC, whereas an ORR of up to 71% has been reported for patients with CC RCC [2,6-11].

### Use of Patient-Reported Outcomes

Throughout several decades, patients’ symptoms and side effects have been assessed by clinicians during consultations. A lexicon—the Common Terminology Criteria for Adverse Events (CTCAE)—that is used to report and grade adverse events was developed in relation to pharmaceutical development, and it is used by clinicians in both clinical trials and daily routines. To date, the CTCAE is the foundation of symptom scoring for patients during active oncological treatment [12]. Nonetheless, research shows a discrepancy between symptoms scored by patients and clinicians where clinicians underscore the severity of the patients’ symptoms [13,14]. Safety and toxicity reports are crucial in clinical trials and must be reliable, making this discrepancy problematic. Since 2009, the incorporation of patient-reported outcomes (PROs) in pharmaceutical research has been a part of guidelines and recommendations [15]. Although the collection of PRO data has taken place for decades, often in terms of quality of life data from patients participating in clinical trials, these data were not being used during data collection, and the focus became benefitting populations of patients instead of directly benefitting the individuals who shared such information. This is called the *passive use of PROs*.

The active use of PROs is now being implemented to a larger extent, and data are being used in real time to give feedback to patients. Recent studies involving the active use of the PROs of patients treated for metastatic cancer have shown significant benefits for health-related quality of life (HRQoL), median overall survival, and overall survival [16,17]. The use of PROs as end points in clinical studies can be of great importance to patients with rare cancer types, including patients with non-CC RCC, for whom treatment is highly individualized, and evidence based on phase 3 studies is difficult to obtain. This approach allows for the individualization of care for each individual patient. Such information strengthens clinical decision-making, as it can be used for detecting changes in a patient’s condition that would otherwise be overlooked or reported later.

The National Cancer Institute created the PROs Version of the CTCAE (PRO-CTCAE), which includes adverse events that are appropriate for self-reporting [18]. In 2016, the PRO-CTCAE was translated into Danish and validated by Baeksted et al [19].

### Gene Analysis

In the IMmotion150 study [20], it was shown that patients can be divided into groups with either an angiogenic or immune profile, depending on the RNA expression of relevant genes. The study showed that giving patients with a certain profile a treatment that targets the profile had a positive effect on progression-free survival (PFS). These findings have since been validated in the IMmotion151 study [21].

At present day, Danish patients with metastatic non-CC RCC who are fit to receive systemic oncological treatment are offered tivozanib, regardless of their histological subtype, unless the patients have a sarcomatoid component or collecting duct RCC.

The possibilities for individualizing treatment are increasing, as results of gene analyses can be made available within a few weeks via next-generation sequencing. Instead of treating patients with non-CC RCC as 1 homogeneous group, the INDIGO study will investigate whether patients’ future course of treatment can be individualized based on knowledge about the gene alterations in their tumor tissue and via the active use of PROs.

### Hypothesis

Our hypothesis is that basing the choice of first-line treatment on the DNA mutations in and RNA profiles of a heterogeneous patient population will increase the ORR of the total population to 30% (10% has been reported for historical cohorts). To achieve this goal for patients with non-CC RCC, we will give personalized medicine and use electronic PROs (ePROs) actively.

## Methods

### Recruitment

Patients with non-CC RCC or 100% sarcomatoid RCC who have been referred to the Department of Oncology at Copenhagen University Hospital – Herlev and Gentofte to receive first-line systemic treatment for metastatic disease can participate in the study if the inclusion and exclusion criteria are met. Patients from other centers in Denmark will be offered referral to the department for the purpose of participation in the INDIGO study.

The inclusion and exclusion criteria were chosen to mimic the inclusion and exclusion criteria for research on RCC in general. These can be seen in Textbox 1.

**Inclusion and exclusion criteria. CC: clear cell; RCC: renal cell carcinoma.**

**Inclusion criteria**

- Signed informed consent
- The patient must be willing and able to follow the protocol
- Age of ≥18 years
- Inoperable, locally advanced, or metastatic disease with non-CC RCC found to be unsuited for surgery with a curative intent
- Sufficient tissue for DNA analyses (corresponding to 10 slides) and RNA analyses (corresponding to 1000 tumor cells).
- Measurable disease according to the RECIST (Response Evaluation Criteria in Solid Tumors) version 1.1 criteria
- Women must have a negative pregnancy test, not be breastfeeding, or be of nonchildbearing potential (menopausal, hysterectomy, or ovariectomy)
- Women of childbearing potential (<2 years after last menstrual period) and men must use effective contraception (pills, intrauterine device, diaphragm, or condom with spermicide or sterilization) or be sexually abstinent during the treatment with the experiment medicine and up to 7 months after the discontinuation of the medicine
- Karnofsky performance status of ≥70%
- Life expectancy longer than 3 months
- Baseline blood samples for hematology
  Leucocytes: ≥3.0 × 109/L; platelets: ≥100 × 109/L; hemoglobin: ≥6.2 mmol/L
- Biochemistry:
  International normalized ratio of ≤1.5
  Activated partial thromboplastin clotting time of ≤1.5 times the upper limit of normal
  Total bilirubin of ≤1.5 times the upper limit of normal
  Aspartate transaminase and alanine aminotransferase of ≤2.5 times the upper limit of normal for patients without liver metastases and ≤5 times the upper limit of normal for patients with liver metastases
  Estimated glomerular filtration rate of >30 mL/min

**Exclusion criteria**

- Prior systemic treatment for metastatic renal cell carcinoma
- Prior adjuvant treatment with immune-checkpoint inhibitors
- Major surgical procedure, open surgical biopsy, or significant trauma within 28 days prior to treatment initiation
- Serious nonhealing wound, ulcer, or bone fracture
- Autoimmune disease or other condition requiring systemic treatment with either corticosteroids (>10 mg/day of prednisolone or similar) or other immunosuppressive drugs
- Metastases in the central nervous system; the patient must undergo a magnetic resonance imaging scan (preferred) or computed tomography scan of the brain within 28 prior to treatment initiation
- Seizures that cannot be managed with standard medical treatment
- If urine dipstick indicates ≥3+ protein, urine must be collected over a period of 24 hours (must be <3.5 g/day of protein), and if dipstick indicates degree 2 proteinuria, urine must be collected over a period of 24 hours prior to each prescription
- Other malignancy within 5 years (except for curatively treated basal cell carcinoma of the skin and/or cervix carcinoma in situ)
- Uncontrolled hypertension (≥150 mm Hg for systolic blood pressure and/or ≥100 mm Hg for diastolic pressure) despite maximum antihypertensive medical treatment.
- Treatment using other investigational drugs or participation in other studies
- Clinically significant (ie, active) cardiovascular disease, such as cerebrovascular conditions (≤6 months), myocardial infarction (≤6 months), unstable angina, New York Heart Association congestive heart failure (degree 3 or greater), or serious cardiac arrhythmia requiring medical treatment; patients with well-managed atrial fibrillation/atrial flutter may be included
- Previous or current other diseases, metabolic dysfunction, clinical findings on physical examination or clinical laboratory findings that give suspicion of a disease or condition that would contraindicate the use of an investigational drug, or a patient with a high risk of treatment complications
- Patient cases where the investigator finds that patient compliance prevents the safe completion of the treatment

### Design

Our study is a prospective, 4-arm, single-center, phase 2 trial at Copenhagen University Hospital – Herlev and Gentofte, Denmark.

Each enrolled patient will receive oncological treatment based on the results of the genetic analyses of tumor tissue. Next-generation sequencing analyses will be performed with the FoundationOne CDx assay from Foundation Medicine, Inc. The tumor tissue undergo hybridization capture-based targeted sequencing for 324 cancer-related genes; gene alterations; and rearrangements, including microsatellite instability and tumor mutational burden. The RNA analyses will be performed at the hospital’s Department of Pathology to search for an immunogenic or angiogenic RNA profile [20].

**Figure 1:**
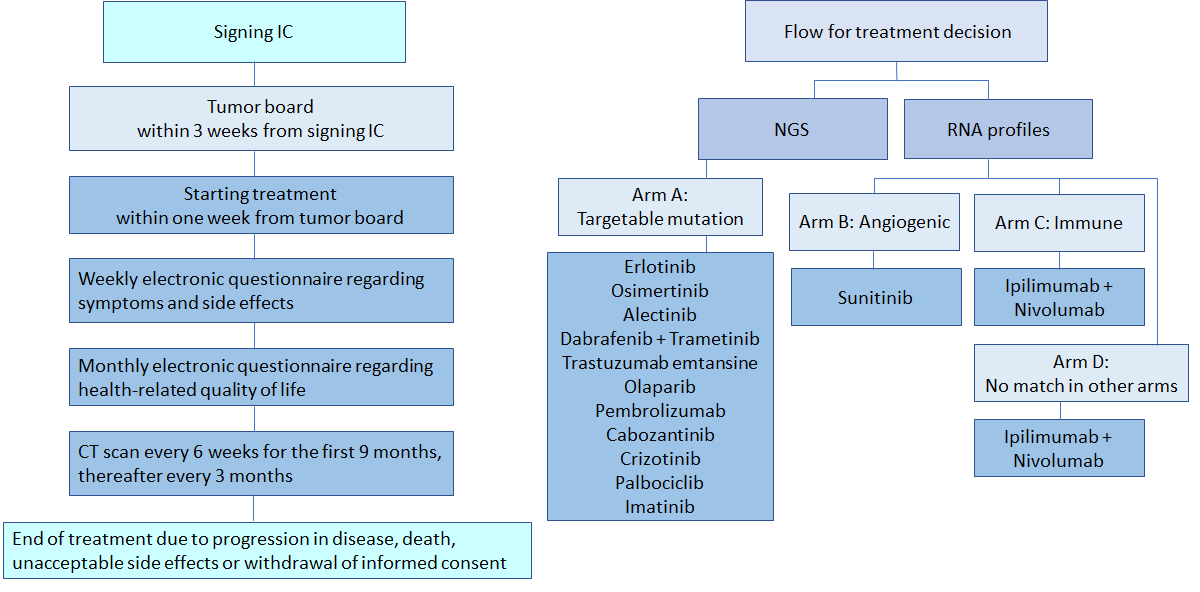
INDIGO study design and flow. CT: computed tomography; IC: informed consent; NGS: next-generation sequencing; RNA: Ribonucleic Acid.

The results of the RNA and DNA analyses will be discussed by a multidisciplinary tumor board, which decides on the treatment offered to a patient. Patients can be allocated to 4 different treatment arms that represent the flow used to allocate the patients. The study flow and treatment arms are shown in Figure 1. If a patient fits into more than 1 arm, the patient will be assigned to the arm that is closest to the first treatment arm—arm A. It is important to emphasize that the design of the study is not to compare the different treatments. The first treatment arm—arm A—is for patients with a targetable mutation, arm B is for patients with an angiogenic profile, arm C is for patients with an immune profile, and arm D is for patients that do not fit into any of the other treatment arms.

Depending on treatment regimen, patients will be seen by a medical doctor every third, fourth, or sixth week to receive treatment. Additionally, the effect of the systemic treatment will be evaluated every sixth week via a computed tomography scan. The patients will receive treatment until clinical or radiological progression, until the patients experience unacceptable toxicity, or until they withdraw their consent to participate in the study.

The intervention in the trial is treatment according to a gene profile combined with the active use of ePROs. Independent of the treatment regimen, the patients will receive 2 electronic questionnaires regarding symptoms, side effects, and HRQoL. At every consultation, the patients’ side effects and symptoms will be discussed and graded by a clinician in accordance with the CTCAE, and a physical examination will be performed.

### The ePROs

All patients will complete a weekly questionnaire consisting of 32 questions regarding 17 symptoms and side effects, which were chosen from the PRO-CTCAE library. The different symptoms are shown in Textbox 2. Regardless of their treatment, the patients will answer the same questionnaires, which use the same advice algorithm. The questions were selected by a group of experts consisting of 4 experts in the oncological treatment of kidney cancer and 2 experts in PROs. The questions were chosen based on the expected frequencies of the symptoms and side effects in all 4 treatment arms and their possible treatments. Rare but potential critical symptoms were also chosen (eg, hemoptysis). The questions were chosen from the validated PRO-CTCAE library and the validated Danish translation of this library.

The questionnaires will be completed in an app, and the patients will immediately receive advice based on their responses. The advice (ie, predefined symptom-handling advice) will be given after an algorithm which is decided on by the expert group. The advice will depend on the severity of the symptoms or side effects reported, and the thresholds for different advice will be decided individually for each question, depending on the symptoms. The questionnaires and advice are in Danish.

The European Organization of Research in Treatment of Cancer (EORTC) Quality of Life Questionnaire-C30 (QLQ-C30) will be completed by the patients every 4 weeks from the start of treatment until the end of treatment. The 4-week schedule was chosen as a compromise among the different treatment regimens (3-, 4-, and 6-week schedule) and due to the possible short participation in the study. The collection of HRQoL questionnaires is an example of the passive use of PROs.

Health care professionals will assess the incoming responses from the patients daily and can contact the patients if necessary. The health care professionals will not receive an alert when a patient has answered a questionnaire, but patients with the most severe symptoms will appear at the top of the list in the clinician interface. At consultation, the PRO responses will be used as a tool and starting point for conversations between the clinicians and patients.

**Figure 2:**
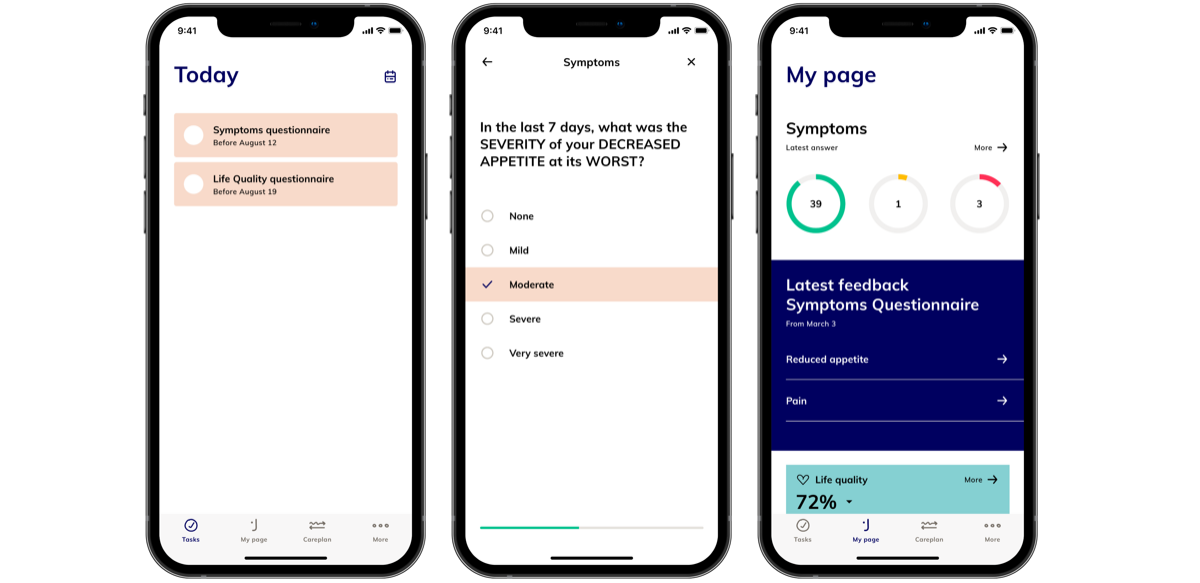
Patient interface in the Journl app. Text has been translated to English.

**Figure 3:**
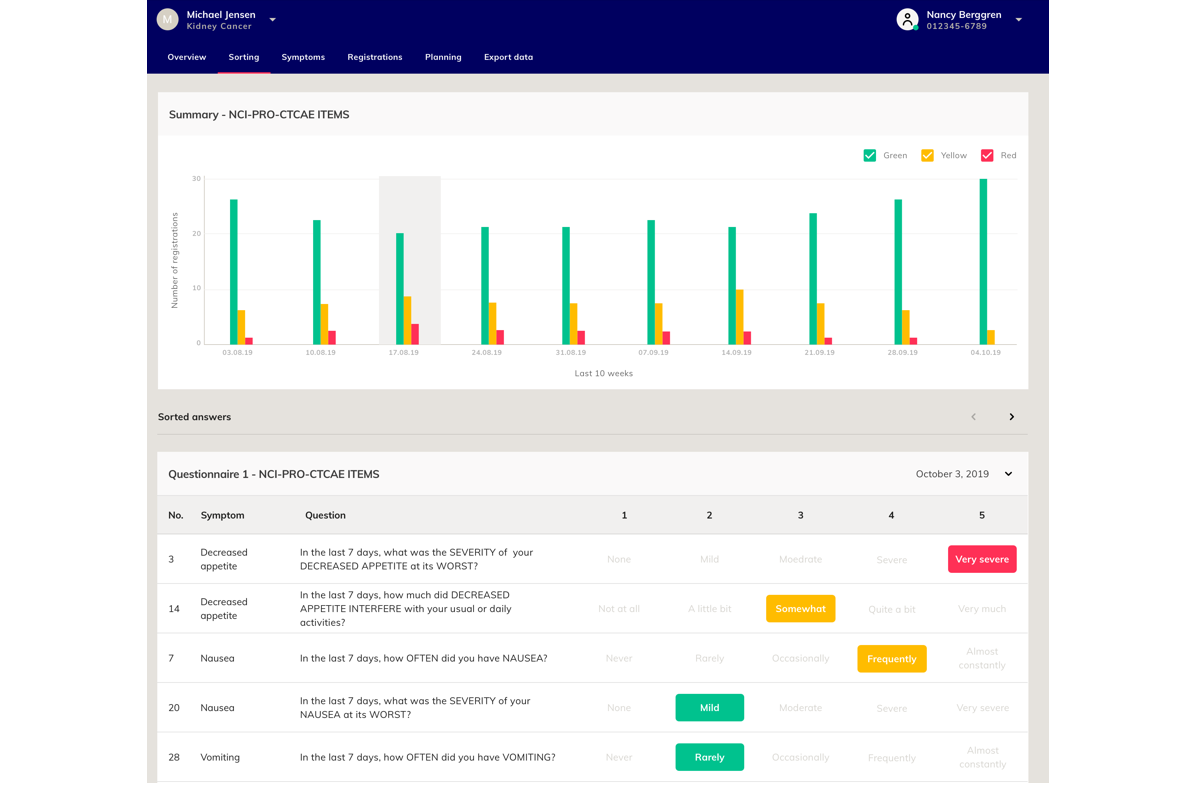
Clinician interface in the Journl web application. Text has been translated to English.

The interfaces for both patients (Figure 2) and clinicians (Figure 3) were specifically developed for the INDIGO study. The questionnaires will be sent to the patients via an app from Journl. Journl is an app provider that specializes in PROs and is International Organization for Standardization (ISO) 13485 and ISO 27001 certified. Before the first treatment, the patients will be instructed to download the app on their smartphones; create a user profile; and complete the first two questionnaires, which represent the baseline. The patients will log onto the app with a personal ID number code. If a patient has uncompleted questionnaires, they will receive daily telephone notifications until the questionnaires are completed. At the end of treatment, the patients will receive a patient-reported experience measure questionnaire in Danish for evaluating patients’ satisfaction with the ePROs [22]. In this questionnaire, the patients will assess, among other things, the length of the questionnaires, whether the questions were understandable, and whether they believed that the questionnaires had a positive influence on their communications with the health care professionals.

**Symptoms in the electronic patient-reported outcomes questionnaire.**

**Symptoms**

- Decreased appetite
- Nausea
- Vomiting
- Diarrhea
- Dyspnea
- Cough
- Pain
- Oral mucositis
- Hypertension
- Fatigue
- Headache
- Dizziness
- Rash
- Pruritus
- Sore muscles
- Coughing blood
- Blood in stool

### End Points

The INDIGO study is a study in which new workflows, including testing new treatment options based on DNA mutations and RNA profiles, and the ePROs of patients with non-CC RCC will be used. The two primary end points are the ORR (complete and partial) in the total patient population, which will be based on the RECIST (Response Evaluation Criteria in Solid Tumors) version 1.1 criteria, and the TTF. The RECIST version 1.1 criteria are standards for evaluating treatment response by measuring changes in the size of tumor tissue. Computed tomography is the preferred modality, and 2 or more target lesions will be chosen at baseline. Afterward, the lesions will be assessed on an ongoing basis. The following secondary end points will be measured: the use of PRO tools during treatment in terms of the completion of weekly questionnaires, in terms of possible patterns in the completion of questionnaires, and in terms of following the instructions regarding contacting the hospital if a patient receives advice to do so. The HRQoL questionnaire (ie, the EORTC QLQ-C30) responses will be converted to values in a graph. Changes in quality of life will be compared with the completion of the patients’ PRO questionnaires. The patients will evaluate their satisfaction with the use of PROs on the validated Patient Feedback Form [22].

Other secondary end points are PFS, overall survival, the disease control rate (ie, complete response, partial response, and stable disease based on the RECIST version 1.1 criteria), the duration of responses, the number of hospital admissions, and the number of adverse events (ie, those in the CTCAE).

### Statistical Analysis

The data will be analyzed with the statistical software R Statistics (R Foundation for Statistical Computing).

Descriptive statistics will be used to describe symptom patterns based on PROs. Further analysis of covariance and 1-tailed *t* tests will be used to estimate changes in selected symptoms and HRQoL from baseline.

Descriptive statistics will also be used to describe patient characteristics and estimate the clinical end points—the ORR, PFS, the TTF, overall survival, the disease control rate, and response duration. PFS, the TTF, overall survival, and response duration will be calculated by using the Kaplan-Meier method. Patients who are alive or have emigrated will be assessed at the end of the follow-up.

Univariate and multivariate analyses will be carried out to assess prognostic factors, based on a Cox proportional hazard regression with 95% CIs. Differences in baseline characteristics will be calculated by using the chi-square test. The case-deletion method will be used in cases of missing laboratory samples, and if possible, multiple imputation will be used.

### Power

When the INDIGO protocol was drafted, the ORR was reported in the range of 10% to 16% for patients with non-CC RCC, whereas an ORR of almost 28% has been reported in patients with CC RCC [2,6]. Due to the high proportion of patients in the International Metastatic RCC Database Consortium poor-risk group in Denmark, the response rates were expected to be closer to 10% [23].

The sample size calculation was based on the following assumptions: if the true ORR is 30%, then at least 30 patients are required to have an 80% probability of demonstrating that the ORR is greater than 10% at a 5% significance level.

The design of the study is not a randomized comparable study.

### Ethics Approval

The INDIGO study has been approved by the National Committee on Health Research Ethics (approval number: H-19041833) and is being conducted in accordance with the Helsinki Declaration. Prior to study-related procedures, the patients must sign an informed consent from and must have received oral and written information about the study. The patients will be informed that they can withdraw their consent at any time without any consequences for their future treatment.

The study has been approved by the Danish Medicines Agency, follows the General Data Protection Regulation, and is registered at the Capital Region of Denmark (ID number: P-2019-232).

## Results

The enrollment of patients started in March 2020, and until June 2022, a total of 9 patients have been included. The inclusion rate has been slower than first expected partly due to the COVID-19 pandemic and fewer patients being diagnosed with non-CC RCC than expected. When the study is completed, the results from the study will be published in international, peer-reviewed journals, and the Vancouver Declaration will be followed in all publications based on the study.

## Discussion

### Principal Findings

The INDIGO study will contribute knowledge about new treatments for a rare and heterogeneous group of patients with non-CC RCC and a poor prognosis. By using personalized medicine based on DNA mutations and gene profiles and by actively using ePROs, we hope to achieve an increase in the ORR. To our knowledge, there are no previous studies regarding treatments based on gene analyses and non-CC RCC or the combination of the active use of PROs and targeted therapy for patients with RCC. The interactive and real-time feedback with ePROs will benefit the patients, since the data will be used to improve the patients’ trajectories. The use of ePROs gives clinicians better insight into patients’ symptoms and side effects in the time between visits to the hospital.

As health care professionals, we should consider whether changing the limits between hospitals and homes (eg, with treatment-related apps on a patient’s private phone) can reduce patients’ quality of life. One can imagine that some patients will find it difficult to maintain their role as a patient when they are at home, while others will see the app, the questionnaires, and the active use of PROs as tools for gaining more influence on their supportive treatment and directing their conversations with health care professionals.

### Comparison to Prior Work

In the INDIGO study, we will use ePROs both actively and passively. The active use of ePRO data can have a great impact on the course of treatment in terms of both the length of treatment and survival. By using ePROs, symptoms and side effects might be detected and treated at an earlier stage before they evolve to an extent that may necessitate a break in treatment or the discontinuation of medication. For some patients, longer treatment will result in longer overall survival. Basch et al [16,17] have shown significant benefits for survival and quality of life in a phase 3 study wherein patients undergoing treatment for metastatic cancer received weekly PRO monitoring. In Basch et al’s [16,17] study, the median overall survival increased by 5.2 months in the PRO group when compared to that of the group receiving standard care, and 1-year survival increased by 6.5% in the PRO group.

Studies that achieve the successful use of ePROs are characterized by automated, severity-dependent patient advice, like those provided based on the alert algorithm in the INDIGO study. The patient advice will guide patients to either contact health care professionals or undergo self-management. Additionally, the health care professionals can access the patients’ reports. In one study, depending on the severity reported, health care professionals had a predefined period in which to react [17,24-26].

### Limitations

First, there are 14 different treatment options, including both immune-checkpoint inhibitors and tyrosine kinase inhibitors, among others, in the INDIGO study. Despite an overlap of side effects among the different treatments, a more specified questionnaire could have been developed for each treatment, which we expect could have been used to detect symptoms earlier. We chose the approach of providing an identical questionnaire to all patients with the expectation that the future will bring more individualized treatment strategies and a demand for a questionnaire that touches on many symptoms without being extensive.

Second, the selection of questions for the PRO questionnaire regarding symptoms and side effects in the INDIGO study is not based on a systematic methodology but is based on an expert group’s assessment. A systematic methodology for the selection of PRO-related questions has previously been described in the literature [27].

Third, the small number of participants in the INDIGO study limits the power of the results regarding ePROs.

### Conclusions

We believe that the addition of the active use of ePROs to the INDIGO study can improve the patients’ trajectories. Our study will contribute further data on personalized medicine for rare types of RCC and provide new knowledge on symptoms reported directly by patients using eHealth tools.

## Abbreviations

CC: clear cell
CTCAE: Common Terminology Criteria for Adverse Events
EORTC: European Organization of Research in Treatment of Cancer
ePRO: electronic patient-reported outcome
HRQoL: health-related quality of life
ISO: International Organization for Standardization
ORR: overall response rate
PFS: progression-free survival
PRO: patient-reported outcome
PRO-CTCAE: Patient-Reported Outcomes Version of the Common Terminology Criteria for Adverse Events
QLQ-C30: Quality of Life Questionnaire-C30
RCC: renal cell carcinoma
RECIST: Response Evaluation Criteria in Solid Tumors
TTF: time to treatment failure

## Data Availability

The study design and the small number of patients participating in the INDIGO study make it impossible to pseudonymize or anonymize data properly. This, in combination with the Danish interpretation of General Data Protection Regulation legislation, makes data sharing infeasible.
